# The Emotional Dimension of Value: A Proposal for Its Quantitative Measurement

**DOI:** 10.3389/fpsyg.2021.807412

**Published:** 2022-02-03

**Authors:** Maite Ruiz-Roqueñi

**Affiliations:** Department of Financial Economics II, ECRI, University of the Basque Country, UPV/EHU, Bilbao, Spain

**Keywords:** stakeholder theory, stakeholder accounting, monetization, social value, emotional value

## Abstract

The first goal of this paper is to develop a theoretical and practical framework which can help to measure the emotional value generated by organizations in quantitative terms. Its second goal is to use data obtained from the UCAN (Union of Food and Agriculture Cooperatives of Navarre) in Spain as a case study to illustrate the quantification of the emotional value generated, with a view to factoring that value into a social accounting system. Ever greater recognition of the social role of organizations in recent years has led to a need for a consistent definition of the concept of socio-emotional value, and for instruments that can be used to measure that value in terms of generic social accounting. Taking the current lack of standardization in such models and instruments, especially those that deal with emotional value as its starting point, the paper proposes a new instrument for measuring that value quantitatively in such a way as to overcome some of the limitations of earlier proposals. The underlying perspective is that the monetary values identified in market and non-market transactions do not accurately account for all the value generated for different stakeholders, and that adjustments are required through a correction factor applied to the value variables identified. The quantification of the socio-emotional value generated by an organization is seen as a more comprehensive indicator of its performance, given that it provides more information and takes into account the value generated for stakeholders as a whole in all dimensions.

## Introduction

There is increasing concern in developed countries at the impact that economic activity is having on society. That concern translates into a greater commitment to society on the part of organizations, through initiatives in the fields of corporate social responsibility (Carroll, 2015; Schwartz, 2017) and, more recently, sustainability (ONU, 2015). More specifically, leading organizations have committed to shifting toward more inclusive business models and incorporating the creation of value for all stakeholders into their business management (Business Roundtable, 2019a,b). Generating value for stakeholders is becoming a key factor for organizations when it comes to legitimizing their activities in the eyes of society (Freeman et al., 2010; Mitchell et al., 2015; Williams, 2018). Institutional initiatives have emerged, such as the Principles of Corporate Government of the OECD and the G20 (OECD, 2016). By establishing principles of policy and governance, they seek to orient organizations toward the design of structures to set and attain goals in regard to their shareholders and stakeholders in general. In this context, organizations that operate in the social economy fit well into these new models of organization (Lazkano and Beraza, 2019; Echanove, 2020) because of their internal values: Prioritizing people and social goals over capital; democratic governability; solidarity; and the reinvestment of most profits in pursuit of sustainable development goals (Social Economy Europe, 2020).

This new, complex setting leads organizations to shift toward finding new ways of generating value for their stakeholders and opens up new research lines associated with the creation of value for stakeholders (Harrison and Van der Laan Smith, 2015; Freeman, 2017; Freudenreich et al., 2020) and with measuring that value (Retolaza et al., 2016; Freeman et al., 2020; Harrison et al., 2020).

Publications on the creation of value for stakeholders are emerging in the field of business management (Harrison et al., 2010; Argandoña, 2011; Harrison and Wicks, 2013; Garriga, 2014; Schneider and Sachs, 2017), but it is necessary to continue obtaining more knowledge and measuring it (Gyrd-Jones and Kornum, 2013; Bapuji et al., 2018; Freeman et al., 2020; Harrison et al., 2020). Few organizations systematically measure the social value that they generate, and there is no single, broadly accepted method for doing so (Olsen and Galimidi, 2008; Tuan, 2008; Mulgan, 2010; Retolaza et al., 2020; Tirado-Valencia et al., 2021).

To help move this line of research forward this paper argues that, from the viewpoint of stakeholders, “value” is better represented by the new logic of “service dominant” value (Vargo and Lusch, 2004, 2008b, 2016; Vargo et al., 2008). The value construct has evolved from an approach in which priority was assigned to the sharing of resources and value was given by price to a new approach in which value emerges from the integration of resources from all stakeholders and is determined by their value in use. The initial approach fits better into orthodox economic models, as it reduces value to economic and financial terms under the point of view of the organization. The new approach is seen from the perspective of the recipient: More dimensions of value are therefore considered, including the emotional dimension. This leaves a gap in the literature in regard to measurement in quantitative terms.

In the face of that problem, this paper seeks to help establish new ways of measuring value generated for stakeholders, and puts forward a proposal on how the emotional dimension of value can be quantified, through a case. So, that proposal is tested at a social organization: UCAN (the Union of Food and Agriculture Cooperatives of Navarre).

The rest of the paper is structured as follows: Section “Conceptual Foundations of the Value Construct and Its Evolution: From Value in Exchange to Value in Use” presents a review of the theoretical framework as regards the value construct, outlining its development and moving toward a more comprehensive conceptualization of stakeholder value. Section “Limitations on the measuring of stakeholder value through financial- Economic accounting” presents the current accounting system used to measure value at organizations, pointing out its limitations in terms of measuring stakeholder value. Section “Socio-Emotional Value” explains why the proposal for measuring emotional value is put forward. Section “Materials and Methods” describes the methodology used. Section “Results” sets out the results as regards the quantification of emotional value at a social organization (UCAN). Finally, a discussion and conclusions are given in the next section.

## Conceptual Foundations of the Value Construct and its Evolution: From Value in Exchange to Value in Use

The creation of value by organizations is a core concept, but one in which there is little agreement as to what “value” actually means or how it is created and distributed or captured (Windsor, 2017).

Axiology is the branch of philosophy that studies the nature of values and evaluative judgments. Reflect on questions such as the nature and classification of values and what kinds of things are or can be valuable. Outside of philosophy, it has a high importance in economic theory (Hirose and Olson, 2015).

The conceptual foundations of “value” and “price” were laid long ago in the field of ethics, where the search for fairness led to efforts to define the fair price of things. The earliest known reflections on value and price are those of Aristotle (384-322 BCE). Those reflections were subsequently consolidated in terms of the notions of value in use and value in exchange. Adam Smith introduced these two concepts with the following explanation: “The word value, it is to be observed, has two different meanings, and sometimes expresses the utility of some particular object, and sometimes the power of purchasing other goods which the possession of that object conveys. The one may be called “value in use” the other, “value in exchange”” (Smith, 1776: 42). In his work he exemplified these two values through a paradox of economic value, explaining that although water is more useful than diamonds, the latter have a higher market price. Years later, as economic science developed, the law of marginal utility and diminishing marginal utility (Krugman and Wells, 2012) solved the paradox of value by establishing that abundance of a good reduces its value in exchange, even if that good has great utility.

The value in exchange has generally been expressed in monetary units and reflected in market prices. Insofar as it is objective and objectifiable, this value subsequently came to be associated with the theory of prices and was developed especially strongly in economics. Other disciplines such as psychology and marketing have focused on value in use, an inherently subjective value determined by individuals. With transactions being the core concept of marketing, value in use became a key idea due to its power to explain why transactions were made voluntarily (Bagozzi, 1975).

Considering the distinction drawn between value in use and value in exchange, the analysis below looks at the evolution of the value construct from the discipline of marketing, due to its greater alignment with stakeholder value.

### Value in Exchange/The Traditional Goods-Dominant Nature of Exchange

The neoclassical economic model argues that economic value is generated in transactions on the market. Under the traditional goods-dominant nature of exchange, the organization manufactures goods and services that are embedded with value. The value generated by organizations and contained intrinsically in goods is exchanged on the market for a sum of money determined by its market price. Economic value is generated because the market price that consumers pay for a good is greater than the cost of the resources used in producing it. The difference between the two values determines the producer’s surplus, which is defined as that part of the value which is captured by the organization or its owners as residual value once the resources used have been paid for Mishan (1968). In this process of voluntary exchange on the market, consumers receive a good for which they pay a market price (value for money). From the viewpoint of consumer stakeholders a consumer’s surplus, i.e., a value, is generated if the maximum price that they are willing to pay for the good based on the utility that it gives them is higher than the actual price paid. The difference between the two is the consumer’s surplus (Dupuit, 1844). In economic terms, the value in use of a good is equated to its utility. This leads to the theory of utility and diminishing marginal utility, which is the theoretical foundation of the value construct (Tellis and Gaeth, 1990).

This influence of neoclassical economics on the way in which consumer value is understood is supplemented by how deeply rooted the concept is in cognitive psychology. The classical definitions of consumer value reflect the cognitive and utilitarian perceptions of value on the part of consumers, and represent it a trade-off between benefits and sacrifices (Day, 1990). One conventional definition of consumer value which reflects this cognitive trade-off is that provided by Zeithaml (1988:14), who defines it as a “consumer’s overall assessment of the utility of a product (or service) based on perceptions of what is received and what is given.” The relaxing of economic logic, maintaining the rationality of individuals in their decisions and decision-making under conditions of perfect competitions and complete information, means that this rational cognitive assessment is based on expected benefits and sacrifices (Bach et al., 1987), which are effectively perceived to materialize once the exchange has taken place. Thus, the difference between expected perceived value and price can be interpreted as the consumer’s incentive to go through with the transaction (Anderson et al., 2009). The value in use perceived is generated in the consumer’s private sphere once the exchange has taken place (Grönroos and Voima, 2013), and it is the consumer who captures or destroys the value contained in the good (Gruen and Hofstetter, 2010).

From this conventional perspective, with its underlying vision of an exchange of product for money, the utility derived from consumption of a good was seen as inherently linked to the physical properties and attributes of that good. Value was thus a value judgment as to the technical quality of the product and the price paid (Cravens et al., 1988; Monroe, 1990).

This initial concept of consumer value is part of a one-dimensional value construct (Sánchez-Fernández and Iniesta-Bonilla, 2007), i.e., a single latent feature or construct underlies a set of items (Hattie, 1985), and it can be measured through its valuation by the consumer.

### Value Through Relationships/Extension of the Concept of Value and Its Scope

The conceptual forerunners of the relational vision of value lie mainly in studies by authors from the Nordic School in the fields of industrial marketing (Webster, 1992; Anderson, 1995; Gassenheimer et al., 1998) and services marketing (Gummesson, 1987, 1996; Grönroos, 1994, 1995; Berry, 1995).

These authors questioned the idea that organizations only generate value through transactions on the goods and services markets, and identified the potential of organizations for generating value through relationships forged with consumers (Wilson and Jantrania, 1994; Ravald and Grönroos, 1996).

This raising of the profile of relational assets extended the concept of value, incorporating a new, more interactive, more experiential perspective. Lanning (1998) defines value in terms of the result of the experiences arising from the relationship with an organization, taking competition into consideration. For their part, Ravald and Grönroos (1996) integrate relational value with the utilitarian value provided by goods and services, and see overall value as a combination of the two (total episode value). Lindgreen and Wynstra (2005) distinguish between two research lines: One which focuses on the value of goods and services and the other on relationships.

This new cognitive-affective approach to value, which is rooted in the psychology of consumer behavior, incorporates a new, hedonic component into the concept of value. As stated by Holbrook and Hirschman (1982), up to the 1980s the literature on value focused exclusively on a utilitarian understanding of the concept. The hedonic and utilitarian components the specific development of different multi-dimension all models of value drawn up by various authors. They include Holbrook (1994, 1999), which identifies four dimensions of value: economic, social, hedonic, and altruistic. Wilson and Jantrania (1994) describe three dimensions of value: economic, psychological or behavioral, and strategic components of relationships. Babin et al. (1994) classify the dimensions into utilitarian value (functional, rational, instrumental, and cognitive) and hedonic (affective, experiential, and non-instrumental). Sheth et al. (1991) identify 5 dimensions: social, emotional, functional, epistemic, and conditional value. This last model was subsequently adapted by various authors in particular contexts (Sweeney and Soutar, 2001; Pura, 2005). Batra and Ahtola (1991) incorporate further components, other than the hedonic and the utilitarian, which they call “thinking and feeling.”

Although these models have the advantage that they are closer to the true complexity of the value construct from the viewpoint of the consumer stakeholder, given that they incorporate intangible, intrinsic, emotional factors, there does not seem to be any agreement among researchers as to what dimensions should be considered as forming part of value or as to how it should be measured. However, assuming that both hedonic and utilitarian components must be taken into account as attitude components, it is clear that an affective component of emotional value needs to be incorporated into the value construct (Lemmink et al., 1998).

The relational perspective focused initially on customer/supplier relationships and subsequently broadened its focus to consider the potential for generating value arising from different stakeholders in their interactions in networks (Payne et al., 2001; Payne and Frow, 2017). The forerunners of this more global take on the relational perspective argued that organizations cannot meet the needs of their clients and maintain stable relationships with them if their cooperation with other parties involved is not equally sound and based on the same relationship principles. This broadens the relational network to all the various actors involved, in a multi-stakeholder approach (Christopher et al., 1991; Kotler, 1992; Gummesson, 1996).

### Value in Use/Service Dominant Logic

The traditional concept of value was based on good-dominant logic, in which value is contained in the product itself or in the relational assets and the ability to create value, and indeed the responsibility for doing so, falls to organizations, which capture part of that value through price. However, more recent concept of value given active role to consumers and other stakeholders which involves them in the value creation process. Competitive logic (Prahalad and Ramaswamy, 2004), service-science (Maglio and Spohrer, 2008; Spohrer et al., 2008), and service-dominant logic (Vargo and Lusch, 2004, 2008a,^2016^) share the approach of placing consumers (or other stakeholders) at the heart of the value creation process, as the main actor(s) in that process. Of all these approaches, the last has probably enjoyed greatest development in terms of a multi-stakeholder perspective.

Service-dominant logic as an alternative theoretical framework for explaining the creation of value is guided by the basic principle that “service” (the term is used in the singular to distinguish it from goods/services), understood as the application of knowledge and skills for the benefit of a third party, is the basis of all exchanges in the economy (Vargo and Lusch, 2004). Reciprocal exchanges of “service” for “service” (knowledge and skills) to the benefit of a third party and the integration of the resources of each party give rise to the creation of joint value, or the joint creation of value (Vargo and Lusch, 2008b,^2009^, ^2014^). From this perspective, stakeholders take part in the process, given that their resources and the application of their knowledge and skills for the benefit of a third party benefit them in terms by giving them access to the resources and applied knowledge and skills of others, since the exchanges involved are reciprocal. This helps them attain their goals (Macdonald et al., 2016; Ranjan and Read, 2016). By developing this logic Vargo and Lusch (2016:7) see joint-creation as “a process where actors are involved in resource integration and service exchange, enabled and constrained by endogenously generated institutions and institutional arrangements, establishing nested and interlocking service ecosystems of actors.”

In this paradigm shift, value ceases to be created and determined mainly by organizations and comes to be jointly created. This means that value is created jointly by multiple stakeholders who cooperate in the process (Bhattacharya and Korschun, 2008; Vargo, 2009; Voima et al., 2010, 2011; Frow and Payne, 2011; Grönroos, 2011). Value is created in networks and not just the consumer but all the stakeholders involved in jointly creating it take part in the exchange and integration of resources, with each contributing to the welfare of the others (Vargo and Lusch, 2016). By contrast with the conventional logic of the exchange of goods, service-dominant logic focuses exchanges in terms of value and extends the prospective, going beyond a dyadic vision (consumer/provider) to form a multi-stakeholder perspective (Vargo and Lusch, 2008a; Vargo et al., 2008). This vision, which stresses cooperation between different actors in the process of joint creation, had already been posited in the relational perspective (Sheth and Parvatiyar, 1995) and backed up by other approaches such as the value constellation approach (Norman and Ramírez, 1994), the network perspective (Hakansson and Snehota, 1995), and service science (Maglio and Spohrer, 2008). A close concept is the shared value creation proposed by Porter and Kramer (2011). This value proposition is very close to the stakeholder approach and quite consistent with cooperative principles and values.

Another major difference between service-dominant logic and the more conventional perspective is the priority given to value-in-use (Vargo and Lusch, 2004), in detriment to the importance previously attributed to value in exchange. Value-in-use started out as a utilitarian approach to value which arises for users during the process of consumption, but the current notion of value-in-use stems from idea that value is jointly created through the integration of operand and operant resources and the exchange of service. Value-in-use does not depend exclusively on the knowledge, skills and resources of an organization but on those of all the stakeholders involved in the value creation process and in the individual way in which they are integrated through activities and interactions for the joint creation of value (McColl-Kennedy et al., 2012).

With this perspective of value-in-use, Vargo and Lusch (2008b) find that value is always uniquely and phenomenologically determined by the beneficiary. Value is idiosyncratic and therefore unique to and determined by each individual. This goes beyond the concept of the intrinsic value of products and services determined by the qualities and/or properties that enable them to meet needs. It is therefore not predetermined in the exchange process, but jointly created in use by all the stakeholders in the system (Vargo and Lusch, 2016). By contrast with earlier concepts of value, Macdonald et al. (2016) argue that value does not emerge exclusively in the process of use that can be experienced throughout the process of relationships and interactions.

The use of the term “value-in-use” by some authors has changed, in the belief that the concept of value-in-context better reflects the way in which value should be understood in the context of the beneficiary’s world and emphasizing that aspect (Chandler and Vargo, 2011).

Value creation has been seen as a process that increases the welfare of beneficiaries and benefits them in some way (Grönroos, 2008; cf. Vargo et al., 2008; Nordin et al., 2011), but it must be considered that the value creation process could also take a negative direction if value is destroyed rather than created (e.g., Echeverri and Skålén, 2011).

The above points reveal that the value construct has evolved substantially over the past few decades. Although part of the focus of the research arises in order to explore and explain consumer value, the concept of co-creation makes it possible to explain the creation of value as a broader and more participatory process that accommodates the different stakeholders (Ind and Coates, 2013; Alves et al., 2016). This view of creating value through the participation and interaction of different stakeholders can find adherence in the stakeholder literature (view Table 1).

**TABLE 1:** From a perspective of value-in-exchange to one of value-in-use.

Value in exchange perspective/Goods-dominant nature of exchange	Value in use perspective/Service-dominant logic
• Value is created by organizations, which determine it • Value is contained in products/services • Organizations delivers value • Organizations capture part of the value created through the price of the goods and services that they provide • Concept of value: trade-off between benefits and sacrifices • Dyadic exchanges (consumer/provider) • Consumer is passive recipient of value • Underlying utilitarian and economic perspective • Roots in economic theory and cognitive psychology • One-dimensional value construct • Market transactions are the key factor • The emotional dimension of value is not taken into account • Prevalence of value-in-exchange	• Value is jointly created through the integration of resources and the exchange of service. Reciprocal. • Value is determined by the beneficiary and experienced in use. • Value creation is the responsibility of the consumer, the provider and other stakeholders in the system, who partly control the process. • Phenomenological perception of value. • Multi-stakeholder perspective. • Underlying cognitive-affective approach. • Roots in behavioral economics and consumer-behavior psychology. • Multidimensional value construct with utilitarian and hedonic components (emotional dimension). • The service for service exchange and the integration of resources are the key factor. • Prevalence of value-in-use.

*Source: own work.*

The evolution of the value construct provides a better, deeper understanding of it and should therefore have an impact in both industry and the academic world, in terms of how value is measured and quantified. Based on these ideas, the next section discusses economic and financial accounting and posits that it provides insufficient information from the viewpoint of value with various beneficiaries (stakeholders).

## Limitations on the Measuring of Stakeholder Value Through Financial- Economic Accounting

Effectively measuring the value that emerges from an organization *via* its relationships and interactions with its various stakeholders is a critical area of management. There seems to be widespread agreement that the management of organizations should be directed generally toward creating value, but when it comes to turning that generic assertion into specifics there is no consensus on points such as: the perspective from which the value should be studied (owners, consumers, employees, stakeholders, society, nations, etc), who creates it, what exactly value is, what is valuable, where value lies, how it should be quantified, who values what, and even whether value should be interpreted in terms of content or as a process of new value creation. We reflect on these aspects below.

The phenomenon of value creation by organizations can be explained in part in terms of market transactions. Classical economics provides tools for analyzing and quantifying that value, and accounting measurements are an essential part of the process of monetizing the value created by any organization (International Accounting Standard Board [IASB], 2018).

Economic and financial accounting acknowledges, measures, and presents information on the transactions carried out by an organization. Making accounting measurements requires two things: A unit of measurement and a model of measurement. The unit of measurement is currency, about which there is very little argument. However, the choice of a model of measurement has been the subject of debate throughout the development of accounting theory. Preferences have shifted from a model based on historical cost to the fair-value-based model that currently enjoys the broadest acceptance among accounting rule-makers in the international setting. Thus, International Financial Reporting Standards indicate that “fair value” is the price received on selling an asset, or the price to be paid to transfer a liability, in an orderly transaction between market participants on the date of measurement (International Accounting Standard Board [IASB], 2011).

The discussions concerning the validity of the current model of measurement go beyond technical arguments and into the field of epistemology, which takes them well beyond the scope of this paper. However, in the interests of research it is worth pointing out that the factors underlying the choice of a model of measurement include who is the target of the information and what business model companies use. According to the Conceptual Framework for Financial Reporting (International Accounting Standard Board [IASB], 2018), the group that makes most use of financial statements is that of investors, lenders, and other current and potential creditors. The information reported must therefore be useful to those users in the sense that it must give a true picture of the economic and financial circumstances of organizations. However, it is worth pointing out that those circumstances are themselves merely a representation of the underlying business model, which is linked reciprocally with the model of measurement chosen. The fair value model rests on a number of assumptions in line with economic orthodoxy as set out in IFRS 13 (International Accounting Standard Board [IASB], 2011), which move it away from the necessary requirements to support accounting for stakeholders.

In terms of the goal of assessing and quantifying stakeholder value, economic and financial accounting seems to have various limitations as a form of measurement.

(1) It reduces value to that which is generated from market transactions, and fails to consider non-market transactions where payment does not take an explicit monetary form. Nor does it consider the fact that exchanges of value are not necessarily limited to goods, services, and cash but may include time, energy, and emotions, or the fact that there may be networking.
(2) It expresses value solely in economic and financial terms. If it is accepted that both hedonic and utilitarian considerations need to be taken into account as components of attitude, in the value construct, then an affective component of emotional value needs to be factored in and considered in valuation.
(3) It directs the information reported at a single stakeholder group. The concept of organizations as entities that generate value through and for all their stakeholders (Freeman, 1984; Freeman et al., 2010; Harrison et al., 2020) requires the value received by each stakeholder to be identified. Value must thus be understood and measured from the perspective of the various parties that benefit from it.

There is no denying that economic value is a fundamental dimension in the measuring of value, or that financial/economic accounting is a good instrument for that purpose. However, given its value-centered perspective, economic and financial accounting does not seem to be a suitable way of expressing stakeholder value, because it suffers from a lack of alignment in terms of both the perspective from which value is studied and the items and dimensions that must be taken into account.

To make up for this lack of information, organizations supplement economic information with sustainability reports, such as the Global Reporting Initiative, integrated reports, SA8000 (Hughen et al., 2014; Morioka et al., 2016) or through accounting systems social (Gray, 2001; Richmond et al., 2003; Gray et al., 2014). However, these systems also have limitations in presenting information qualitatively in some cases and/or not taking into account the emotional dimension of the value.

## Socio-Emotional Value

Given the points set out above and the fact that there is no place for the emotional dimension of value in current value measurement systems, this section proposes a way of measuring it. Out of the various systems available in social accounting, we have opted for an integrated social value model (Retolaza et al., 2016), given that such models have been proven to be applicable to organizations of all types (Retolaza and San-Jose, 2021).

This model is based on four assumptions: Action research, stakeholder theory, the phenomenological perspective and fair value based on fuzzy logic. For more details, see publications that discuss social accounting (Retolaza et al., 2013, 2014, 2015, 2016).

The social accounting model seeks to provide conceptual support which is sound enough to enable progress to be made toward more comprehensive measurements of value. It thus enables the various categories or ecosystems of value identified to date to be brought to light. Those categories are outlined below.

### Market Social Value

This is an initial approach to measuring social value and its distribution. It is calculated as per the proposal set out by Gonzalo and Pérez (2017), using value added analysis, and by the GRI4 (Global Reporting Initiative [GRI], 2013) in regard to value created and distributed. It reflects the social value that arises from market transactions between a company, its clients, the public administration, and other resource providers. It is an improvement on the information provided by organizations concerning value distributed to their various stakeholders, but the value identified is solely financial and restricted to that which arises from transactions on the market. Despite its narrow vision of stakeholder groups and value made visible, it has the advantage of making it easier to obtain the information needed for calculations, given that it is based on data already identified and collected by organizations, which can be found in their accounting records.

In general, this analysis takes on board the value creation principles of the neoclassical perspective. The creation of value is independent from its distribution. Value creation is focused on generating surpluses for owners (shareholders). Value for other stakeholders is brought to light by applying the shareholder’s financial value perspective to stakeholders as a whole, without making any broader interpretation of value, perhaps from the perspective of stakeholders themselves.

### Non-market Social Value

This is another category or ecosystem of value, and as such it can help bring to light social value more accurately. It identifies the social value that arises from transactions between the company and its stakeholders which are not made on the market. It is particularly useful for organizations where the value generated and distributed comes mainly from non-market mechanisms (Sandel, 2012).

It is measured from the perspective of stakeholders, i.e., the groups of individuals and organizations for which an organization generates or may generate value. From a phenomenological perspective, it is stakeholders who identify the sources or variables of value, so dialog with the various stakeholders is required to obtain the information needed. In this case there is often no market value, so value variables expressed in general terms need to be reformulated as indicators associated with outputs measurable by the organization. Once the relevant proxies have been selected, those outputs can be measured in monetary units.

### Emotional Value

When the social accounting model was initially developed (Retolaza et al., 2016) it identified this category of value and noted its importance, but the emotional dimension of value was not quantified because it required a different measuring procedure, given that it was not only subjective but also intangible. This led to it initially been considered for my descriptive, covering the circumstances and emotions of the various stakeholders. However, the qualitative nature of the information collected made it difficult to integrate into the other categories identified and valued in monetary terms. However, despite the difficulties of its measurement, the entities considered that said value should be taken into consideration if it was desired to identify the totality of the perceived value, in all its dimensions and forms. What is measured is managed (Kaplan and Norton, 1992) and if a complete and exhaustive measurement of the value generated and distributed to the stakeholders was not carried out, it could hardly be managed as a whole. Incomplete measurement of socio-emotional value, ignoring emotional value, could act as a deterrent to implement management approaches centered on persons or on social approaches.

To make progress in the assessment and quantification of emotional value and in integrating it with the other categories in the model, a proposal for measuring it was put forward at a summer course at the University of the Basque Country (UPV/EHU) (Retolaza and Ruiz-Roqueñi, 2018) and subsequently published (Ruiz-Roqueñi, 2020) in the special issue of the *CIRIEC Journal* in 2020 (“Special Issue n°100 about Social Accounting, 2020”).

This proposal consists of using an index that operates as a correcting factor, enabling market and non-market social value already calculated to be adjusted up or down (Guasch, 2015). The underlying perspective is that monetary values already identified in market and non-market transactions do not accurately reflect the full value generated for the various stakeholders. For example, the value that two specialists get from their relationship with an organization may differ depending on the emotions generated by their interactions with it, even though the market social value quantified in terms of net salary may be identical for them both. Thus, although the market social value received by the two specialists with identical salaries is the same, to establish the experienced value a correction factor would need to be applied to adjust and salary up or down depending on their experiences. The final outcome of this measure of value reflects the duality of human beings (utilitarianism/hedonism).

The correction factor could be: (1) unique and applicable to the full, aggregate market and non-market social; and (2) multiple, which would entail identifying a correction factor for each stakeholder group, or more precisely identifying a separate correction factor for each value variables identified by each stakeholder group.

The scale of measurement of the SERVQUAL model (Parasuraman et al., 1988, 1991) adapted to each organization was used to obtain correction factors. As well as identifying the factor(s) involved, a range must be set (upper and lower bounds) so that the extent to which the factor corrects the value can be specified. To calculate this, an adapted questionnaire was designed using the SERVQUAL scale, with an additional question to identify the weight of emotional value relative to other values, with a view to determining the range (Ruiz-Roqueñi, 2020).

The information needed to calculate the correction factor and the range must be provided by a representative sample of the population under study. In this case, that means the recipients of the value, i.e., stakeholders. A representative sample of stakeholders needs to be used for the results to be statistically reliable.

As an improvement on this proposal, we propose here that the Net Promoter Score (NPS) developed by Reichheld (2003, 2004) and consultancy firm Bain & Company be used as an alternative to the SERVQUAL model to calculate the correction factor to be applied to each value variable identified and each group of stakeholders.

The two measurements are based on different constructs in terms of standard of service and satisfaction (Bolton and Drew, 1991; Cronin and Taylor, 1992; Bansal and Taylor, 1999). But they are linked and the literature contains many publications in which these two concepts are used as synonyms. Thus, the links between the standard of service construct and satisfaction are not yet clear. Some studies see two-way link between them (Oliver, 1993; Iacobucci et al., 1994) and others find one-way link (Bitner, 1990; Patterson and Johnson, 1993). The fact that the dominant theory behind their conceptualization is the same in both cases (the disconfirmation paradigm) helps to establish similarities between them (Day, 1977; Swan and Martin, 1981). They both consider the viewpoint of clients as a core idea. The literature also recognizes that both concepts stem from the performance of the service being measured against a standard, i.e., predictive expectations in the case of satisfaction and the desired standard of service in the case of quality. So although they are different, the similarities between the two concepts make them suitable for use in the goal pursued here of incorporating the hedonic dimension of value not reflected in market and non-market social value measurements, given that both constructs contain both affective and cognitive components.

Our proposal is motivated, however, by technical, and especially operational, considerations. By contrast with the SERVQUAL model, which is conceptualized as a multidimensional construct of standard of service, NPS is a tool for measuring satisfaction and loyalty. It is designed as a one-dimensional construct that varies on a continuous line from unfavorable to favorable or from zero to high in terms of probability of recommendation. This makes it simpler to use than the previous model. One of the criticisms leveled at the SERVQUAL model is that it is hard to implement. Multidimensional constructs provide a holistic representation of a complex phenomenon, but their main drawback is that they are hard to implement in practice. By contrast, the Net Promoter Score (NPS) has been broadly used by managers at a great many businesses, and over time has come to be used for different purposes, e.g., as part of the performance measurement system at organizations (Faltejsková et al., 2016).

### Socio-Emotional Value

The adding together of the values obtained in different categories or ecosystems of value is referred to as “socio-emotional value.” It incorporates three different types of value measured with a single unit of measurement.

## Materials and Methods

The study focuses on a single intervention, using the case study method (Yin, 2014). This method was chosen for its proven effectiveness in developing new theoretical and practical knowledge which is checkable and can be empirically validated (Eisenhardt and Graebner, 2007).

The organization selected as a case study for measuring emotional value was UCAN. This organization has 126 members and has been in existence for over a century. Its corporate purposes include “promoting and organizing services and actions of general interest to farming cooperatives, to cater for the needs of and foster development and effectiveness in the management of food and agriculture cooperatives” (UCAN, 2021).

Union of Food and Agriculture Cooperatives of Navarre was chosen for several reasons: It is medium-sized and its area of operation is Navarre, which made it easier for us to contact the organization and its stakeholders. Secondly, there was its track record: this structure of food and agriculture cooperatives in Navarre is a benchmark throughout Spain. Thirdly, a key element for the success of the project was that the management of the organization became closely involved in it. This was essential for the practical implementation of the method for measuring social value at the organization. Finally, UCAN had been using an integrated social value social accounting model since 2016 (Retolaza et al., 2016) to qualify shareholder value. Even more importantly, it had already calculated emotional value in monetary terms in that year.

The ongoing pursuit of excellence spurs the management of the organization to explore new measures for successful operation and demonstrate to stakeholders that it makes a contribution to society, as required under its founding purposes. The interests of the management therefore coincided with those of this investigation, which seeks to validate the model in terms of practical applicability. Identifying any practical problems that might arise in its implementation and detecting and implementing potential improvements enables the model to become consolidated and validated, with a view to extending its use to organizations of other types.

Socio-emotional value was measured in three phases.

(1) Identification of market social value. The market value is obtained from the transfer of the information contained in the profit and loss statement to determine the value created and distributed to the different stakeholders from market transactions between a company and its stakeholder groups. This value also incorporates the value generated in transactions through purchases from suppliers.
(2) Calculation of non-market social value. The social accounting model measures this *via* a six-step process: (1) setting up of a team and approval of a timeline; (2) identification of the stakeholders for whom the organization is assumed to generate value; (3) identification of value variables, in the sense of points in which the organization generates value for third parties; (4) intersubjective quantification of the outputs linked to each value variable through proxies; (5) integration of results to give what we refer to as the integrated social value; and (6) feedback and continuous improvement.
(3) Measuring of emotional value. It is the value determined and experienced by the person in their relationship with an entity and that is not included in the market price or fair value. It can come from sources such as: satisfaction of the stakeholders in their relationship with the entity, security offered by the entity, or in the case of agricultural cooperatives due to their impact on actions to stop depopulation, make visible the role of farmer and rancher or the conservation and maintenance of the land, among others.

Each phase of the process requires different, specific information. The basis for the calculations in phase 1 comprised the financial and economic information reported by the organization and accounting information from its suppliers, obtained *via* the SABI database.

In phase 2 much of the information required was obtained through interviews. Interviews are an effective research tool when a complex analysis using detailed information is required. They also create a relaxed atmosphere that is conducive to data collection (Patton, 2002) and are particularly useful when tackling problems that require in-depth study. Twenty-one interviews were held with representative members of each stakeholder group, between February and March 2020.

The information for phase 3 came from questionnaires. Information representative of each stakeholder needed to be obtained so, given the size of the study population, questionnaires were sent to everyone in it between December 15, 2020 and January 15, 2021. The answers obtained allowed reaching a level of confidence higher than 80%, with a margin of error of 5%.

## Results

The goal was to express the emotional value of UCAN in monetary terms, so first of all the social value generated by the organization’s market and non-market operations was calculated, with a view to determining its integrated social value per annum. Once this figure was obtained, the correction factor was applied to each variable identified in the integrated social value to obtain the emotional value.

The market social value of UCAN was calculated on the basis of financial performance data, which in this case means wages, social security contributions, taxes, withholdings and profit/loss for the year. With that data, it was found that UCAN generated a value for society (stakeholders) of €299.814 from direct market transactions in 2020, with returns to the public administration of €122.743. The organization’s suppliers were analyzed in the same way, and the amount in indirect market social value attributable to UCAN was taken as the relative value of their invoicing. Thus, the social value generated indirectly through purchases from suppliers was €162.657, and the returns for the public administration were €82.389.

After all the relevant steps had been performed, the non-market social value was calculated to be €2424883.

The market social value generated (€462471) was integrated with the non-market social value (€2424883) to give the integrated social value generated by the organization. For UCAN (2021), this resulted in a figure of €2887354.

Emotional value was calculated by applying a correction factor obtained from the NPS to each value variable and each stakeholder group identified in the previous phase, this resulted in a figure of €2298633. The correction factor is therefore not unique, but rather a separate correction factor is applied for each stakeholder group and for each value variable.

The results for socio-emotional value, broken down by categories or ecosystems of value and their distribution across the different stakeholder groups of UCAN (2021) are shown in the following table (view Table 2).

**TABLE 2:** Socio-emotional value.

	Society	Public administration	Suppliers	Employees	Investors	Social organizations	Members
Market social value	€462471	€205131	€150164	€230892	€9185		€299814
Non-market social value	€2424883	€161113	€5715	€636		€180982,5	€2322083
Integrated social value	€2887354	€366244	€140251	€231528	€9185	€180982,5	€2621897
Emotional value	€2298633	€16040				€12778	€2269815
Socio-emotional value	€ 5185987	€ 382.284	€140251	€231528	€9185	€ 193760,50	€ 4891712

*Source: own work.*

## Discussion and Conclusion

This study looks at how the meaning of the concept of value has changed over time from an economics-based respective represented by value in exchange through a transition phase to a new perspective characterized by value in use or contextual value. That evolution of the concept of value is reflected, albeit independently, in the way in which value is measured, and this has various implications.

Firstly, it is worth highlighting that under the value-in-use approach (given that value is jointly created in the context of a value ecosystem involving various actors), the measuring of value entails identifying the value in use of each actor. This means a shift toward a multi-stakeholder perspective of value. In the classical economics-based approach, value is created mainly by organizations.

Secondly, increasing knowledge of the concept of value has enabled at least one more component to be incorporated into the value construct: the hedonic component. In combination with the classical, utilitarian component this provides a better, more comprehensive vision of value. The narrow perspective of value in classical economics, where there is no place for the emotional dimension, contrasts with this new perspective in which the emotional dimension is valuable element, so tools for measuring it are needed.

Thirdly, there is no denying that economic value is a fundamental dimension in the measuring of value, or that financial/economic accounting is a good instrument for that purpose. However, that instrument seems to suffer from limitations when it comes to measuring and quantifying stakeholder value, so it would be useful to develop an accounting system for quantifying stakeholder value and all its dimensions, including emotional value.

Fourthly, the social accounting model used [that of integrated social value (Retolaza et al., 2016)] extends conventional ways of measuring performance at organizations and highlights both the generation of value and its distribution. This makes it possible to reflect on the effects of performance measurement at organizations on two levels: (1) in terms of the excellence (or failure) of management; and (2) in terms of how far it contributes to or hampers the welfare of stakeholders.

The practical implementation of the model proposed at UCAN provides a more comprehensive vision of the value generated by that organization. The data obtained reveals that part of the value emerging from the organization is not shown in economic/financial accounting. UCAN is a social organization, so much of the value that it generates is not reflected in market transactions. Finally, the data shows that most of the emotional value created comes from its interactions with its members. It is true to say that the concept of emotional value applies to all stakeholder groups, but it is particularly significant for those which participate in and are involved with the organization most strongly.

From a management viewpoint, this study makes two contributions.

First, it helps to develop social accounting by providing a new, more complete tool for measuring value that incorporates the measuring of its emotional dimension. This enables managers to measure emotional value in a relatively simple fashion, so that it can subsequently be included along with other value ecosystems in a single, monetary unit of measurement.

Second, it extends the possible ways of measuring performance at organizations. This provides more comprehensive information on the value that they generate, organized into three value ecosystems (market value, non-market value and emotional value). That information can be used by managers for communication, strategy and control purposes. It also provides information to the various actors involved to confirm the contribution of each organization to the attainment of overall social goals, by creating greater welfare for stakeholders as a whole within the value generation system of each organization.

The design of tools for measuring emotional value is still at a very early stage, so the proposal set out here must be seen in the context of exploratory research. Thus, the limitations of the study can be attributed largely to the inductive design of the model and to the fact that it analyses only a single case study. The implementation of the emotional value measuring tool at UCAN confirms its applicability from a practical viewpoint. However, it remains to be seen whether the use of the tool can be extended to organizations of all types, and how suitable the value correction factor chosen is.

The practical approach taken in this study means that the choice of value quantification instruments was limited by cost factors and by the resources required for measuring, without prejudice to the rigor of the analysis conducted. Future research could work on improving the model. Accordingly, reputation could be considered as a correction factor. Specific analyses of the link between financial/economic performance and social performance suggest that reputation could be a significant mediating factor in a positive link between measurements of these two types of performance (Orlitzky et al., 2003).

Another potential line of future research is the extension of the conceptualization and measuring of emotional value individually for each stakeholder group, through the generation of *ad hoc* value constructs for specific stakeholders, e.g., customers or employees.

Finally, looking at the concept of value in terms of jointly created value opens up new lines of argument in stakeholder theory by admitting the possibility of increases in overall value without collusion with other stakeholders.

## Data Availability Statement

The raw data supporting the conclusions of this article will be made available by the authors, without undue reservation.

## Author Contributions

The author confirms being the sole contributor of this work and has approved it for publication.

## Conflict of Interest

The author declares that the research was conducted in the absence of any commercial or financial relationships that could be construed as a potential conflict of interest.

## Publisher’s Note

All claims expressed in this article are solely those of the authors and do not necessarily represent those of their affiliated organizations, or those of the publisher, the editors and the reviewers. Any product that may be evaluated in this article, or claim that may be made by its manufacturer, is not guaranteed or endorsed by the publisher.
